# mSphere of Influence: Mapping tRNA epitranscriptome by sequencing

**DOI:** 10.1128/msphere.00328-26

**Published:** 2026-07-20

**Authors:** Satoshi Kimura

**Affiliations:** 1 Department of Microbiology and Immunology, Cornell University College of Veterinary Medicine 43317, Ithaca, New York, USA; Virginia-Maryland College of Veterinary Medicine, Blacksburg, Virginia, USA

**Keywords:** tRNA modification, RNA sequencing, molecular methods

## Abstract

Satoshi Kimura works in the field of RNA modification. In this mSphere of Influence article, he reflects on how the paper “Efficient and quantitative high-throughput tRNA sequencing” by G. Zheng, Y. Qin, W. C. Clark, Q. Dai, et al. (Nat. Methods 12:835–837, 2015, https://doi.org/10.1038/nmeth.3478) has impacted him by demonstrating that deep sequencing technology is harnessed to profile tRNA modifications.

## COMMENTARY

“Efficient and quantitative high-throughput tRNA sequencing” established a method for the efficient and quantitative sequencing of tRNAs using next-generation sequencing (1). tRNA is an essential adaptor molecule responsible for codon decoding. Its function and stability are crucial for cellular fitness and survival, and defects in tRNA function can lead to fitness impairments and diseases (2). tRNA is also the most heavily modified RNA species containing a wide variety of chemical modifications. These modifications expand the chemical repertoire of RNA and fine-tune tRNA functions, including codon decoding, amino acid charging, and intracellular stability.

An efficient and quantitative method for profiling tRNA abundance and modifications is essential for understanding how organisms establish their tRNA repertoire, including modification states, to support efficient translation. Such a method would also enable investigation of how organisms dynamically adjust tRNA abundance and modification profiles in response to environmental or cellular changes to optimize their translatome. Because tRNAs are RNA molecules, next-generation sequencing appears to be a natural approach for their analysis. However, sequencing tRNAs has historically been challenging because their extensive modifications and rigid secondary structure interfere with cDNA synthesis by causing reverse transcriptase stalling or termination. This study addresses these technical challenges and establishes a robust approach for high-throughput tRNA sequencing.

The authors implemented two strategies to efficiently sequence tRNAs. First, they used a highly processive reverse transcriptase, thermostable group II intron reverse transcriptase, or TGIRT, for cDNA synthesis. As described above, commonly used reverse transcriptases readily terminate at the modification sites, particularly when a modification disrupts Watson-Crick base pairing between template RNA and incoming dNTPs during cDNA synthesis. The presence of multiple modifications can lead to premature termination of cDNA synthesis and significantly reduce the sequencing coverage in the 5′ end regions of tRNAs. TGIRT is highly processive and can read through many modifications, thereby improving coverage across tRNA molecules. The second strategy was pretreatment of tRNA samples with a demethylase that removes methyl modifications from RNA. A substantial fraction of tRNA modifications are methyl-modifications. *In vitro* treatment with purified demethylase erases many of these modifications and further improves reverse transcription efficiency and sequencing coverage. Together, these two strategies significantly enhanced reverse transcription and enabled efficient and relatively unbiased profiling of tRNA abundance.

Another important highlight of this paper was the demonstration that tRNA sequencing (tRNA-seq) can be used to predict modification sites. tRNA sequencing data contain misincorporation of wrong bases and the termination of reverse transcription. These reverse-transcription-derived (RT-derived) signatures enable the prediction of many modification sites. This observation inspired me to use tRNA-seq to profile tRNA modifications. tRNA modifications are found in all domains of life; however, their distributions and identities have been characterized in only a limited number of model organisms. One reason that tRNA modification profiling has lagged behind is that conventional methods are labor-intensive. Comprehensive profiling requires purification of individual tRNAs, more than 40 tRNA species per organism, followed by analysis using liquid chromatography-mass spectrometry or other approaches. Therefore, I realized that tRNA-seq could serve as an efficient profiling method for tRNA modifications, particularly in uncharacterized organisms.

I began profiling tRNA modifications in *Vibrio cholerae*, the causative agent of cholera, using tRNA-seq and compared its RT-derived signatures with those of *Escherichia coli*, whose tRNA modifications have been extensively characterized. Although these two organisms are phylogenetically closely related, I observed numerous unique RT-derived signatures in *V. cholerae* tRNAs, suggesting the presence of *V. cholerae-*specific modifications. Subsequent mass spec analyses led to the identification of a novel RNA modification, acetylated acp^3^U (acacp^3^U), and a previously undescribed RNA editing process (C-to-Ψ) in *V. cholerae* (3). In this approach, tRNA-seq allowed us to focus on a subset of candidate tRNAs rather than purifying and analyzing all tRNA species. It was exciting to discover such organism-specific modifications because they suggest that individual organisms tailor their tRNA modification profiles to optimize translation. In addition, tRNA-seq can be used to monitor dynamic changes in tRNA modification profiles. Taking advantage of this capability, we recently profiled tRNA modifications across different life stages of *Trypanosoma cruzi*, the causative agent of Chagas disease, and revealed dynamic remodeling of the tRNA modification landscape (4). Among multiple modifications, we identified one modification, OH-yW, that suppresses differentiation from the non-infective form to the infective form. This example highlights the utility of tRNA-seq for investigating changes not only in tRNA abundance but also in tRNA modification profiles.

Many sequencing-based approaches for profiling tRNA abundance and modifications were developed following this pioneering work (5). The study by Zheng G. and Qin Y. opened my eyes to the power of introducing new technologies into the field to address important biological questions.
